# Trend in incidence of gastric adenocarcinoma by tumor location from 1969–2004: *a study in one referral center in Iran*

**DOI:** 10.1186/1746-1596-1-5

**Published:** 2006-05-11

**Authors:** Afshin Abdi-Rad, Siavash Ghaderi-sohi, Hosein Nadimi-Barfroosh, Sara Emami

**Affiliations:** 1 Surgical Pathology Department, Cancer Institute, Tehran University of Medical Sciences, 1497 Keshavarz Blvd., Tehran, Iran

## Abstract

**Aim:**

In recent years several studies have shown increasing rate of *upper gastric *cancers regarding to decrease in distal gastric cancers. The aim of this study was to describe the trend of gastric cancers by location in Iran, which is one of the countries with high prevalence of gastric cancers.

**Methods:**

All registered cases of gasterectomy in Tehran Cancer Institute from 1969 through 2004 were re-evaluated clinicopathologically. The stomach was anatomically divided into the upper, middle, and lower third. The *prevalence *of gastric cancers in *5 year periods *estimated by location and the changes trough the time was evaluated independently and in aspect of age and sex.

**Results:**

Over 36 years, the *prevalence *of cancers in the upper and middle third of the stomach have increased and that of the lower third has decreased. These changes were seen in both sexes and age groups under and over 50 *and it was more significant in younger*.

**Conclusion:**

The results are the same as most previous reports in other countries. This can indicate different risk factors as well as confrontation with them. However in regard to few numbers of cases in this study, a population-based study is recommended for confirmation.

## Introduction

Gastric cancer incidence has markedly decreased in some countries, such as United State [1] but it remains high in others such as Japan [2] and Iran [3,4]. It is the first leading cause of cancer-related deaths in men, and the second one among women in Iran [3]. According to the identification of many predisposing factors in recent years, incidence of gastric cancer has declined in most countries, especially in developed countries [2,5]. Meanwhile, many studies show that adenocarcinomas arising from gastric cardia have increased, especially in areas with low incidence of gastric cancer [6-22]. By contrast, some of the studies have not shown any increase in cardia carcinoma incidence [23,24]. Two important population-based studies indicate that the cardia carcinoma incidence in different countries has been inconsistent [25,26]. There is also a belief that this rising incidence has been confined to the areas with low-risk for gastric carcinoma [23,24], although there are some contradictory studies [19,20,27]. It should be remembered that in spite of a large number of studies carried out in low-risk countries [2], very rare ones have been performed in high-risk countries.

Although Iran is one of the countries with high incidence of gastric cancers, there is no information about possible gastric carcinoma location change in Iranian patients. Therefore, this study intends to evaluate the gastric carcinoma location trend during the last 36 years.

## Materials and methods

As a preliminary step, all cases of gastrectomy specimens due to carcinoma during 1969–2004 in Cancer Institute of Tehran Medical University, the oldest main referral center for cancerous patients in Iran, were collected. All new cases with diagnosis of adenocarcinoma were also included in the course of this study. All the related pathology reports and hematoxylin and eosin sections were re-evaluated and reviewed by two pathologists. In cases with inappropriate slides new sections were cut and stained. Cases with damaged paraffin blocks were excluded from the study.

Anatomic site of each tumor was first determined by referring to the relevant macroscopic description of pathology report and then correlated with the latest guidelines for gastric cancer classification by Japanese Research Society for Gastric Cancer, in which the stomach is anatomically delineated into the upper, middle and lower thirds by dividing the lesser and greater curvature in two equidistant points and joining these points [28]. Tumors located predominantly in the gastro-esophageal junction and cardia were determined to be in the upper third of the stomach, those located in the pylorus were considered to be in the lower third and those located in the mid-body were determined to be in the middle third of the stomach. If tumor was located across adjacent regions, the region containing the greater proportion of the tumor was considered to be the tumor's main location. Cardia was defined as region between 1 cm proximal and 2 cm distal to gastro-esophageal junction [29]. In regard of rather low number of cases per year, each five years categorized in one group and then the trend of location during this 35 years was evaluated.

Clinical data, including sex and age, were gathered from pathologic reports. Demographic and clinical data were analyzed via Pearson's X^2^, ANOVA, and Somer's d. A value of *P *< 0.05 was considered to be statistically significant.

## Results

All 1310 cases of gastrectomy for adenocarcinoma during 1969 to 2004 in Tehran Cancer Institute were collected, from which 78.5%were male. The mean age was 56.6 ± 21.2. In gastric sublocation analysis, 89 cases were excluded because of unavailability of clinical data and 12 cases for multicentricity. Among the rest, 298 (22.7%) were in the upper third, 325 (24.8%) in the middle third and 586 (44.7%) in the lower third. According to lauren classification 54.9% were intestinal type and the remaining was diffuse type.

There was no sexual difference between locations (Pearson chi-square, X^2 ^= 1.47, *P *= 0.479). Analysis of variance showed a significant difference in age at diagnosis between adenocarcinomas appeared in three part of stomach (*P *< 0.001) and in two genders (*P *< 0.006), but no significant correlation between location of tumors and gender (*P *> 0.05).

**Figure 1:**
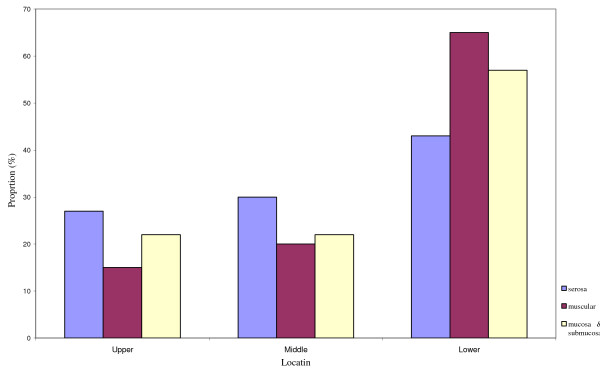
Invasion of gastric adenocarcinoma by tumor location. The serosal invasion was higher in adenocarcinoma of upper and middle third (*P *= 0.000)

There was significant difference between locations and the degree of invasion (Pearson chi-square, X^2 ^= 26.5, *P *= 0.000) which means serosal invasion was higher in adenocarcinoma of upper and middle third (Figure 1).

**Figure 2:**
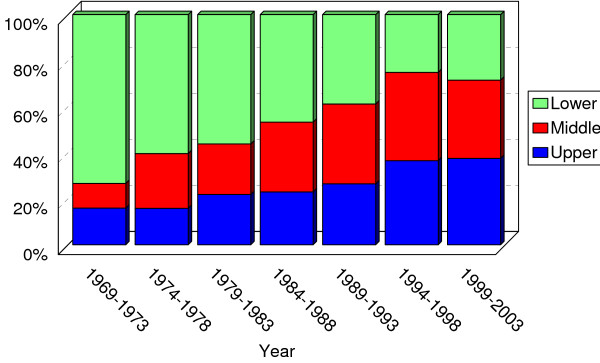
Trend in location of gastric adenocarcinoma. The diagram shows increasing prevalence of adenocarcinomas of upper and middle parts of stomach during recent 35 years (Somer's d = -0.252;*P *< 0.001).

**Figure 3:**
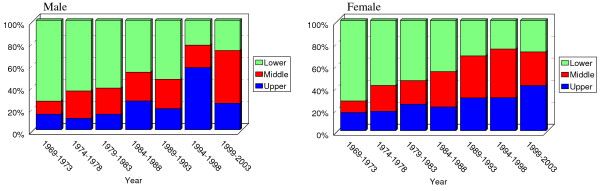
Trend in location of gastric adenocarcinoma in each sex. The increasing prevalence of adenocarcinomas of upper and middle parts of stomach is seen in booth sexes (Somer's d: -0.250 in males and -0.263 in female; both *P *< 0.001)

**Figure 4:**
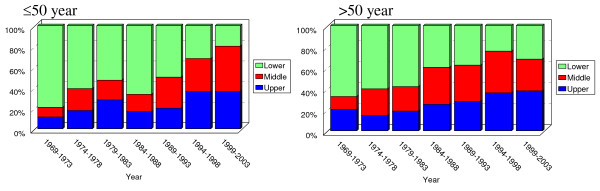
Trend in location of gastric adenocarcinoma by age. The increasing prevalence of adenocarcinomas of upper and middle parts of stomach is seen in booth groups and it is a little more pronounced in younger patients (Somer's d: -0.239 in younger and -0.227 in elders; both *P *< 0.001)

Prevalence of adenocarcinoma of upper and middle third has increased in recent 36 years. By contrast, that of lower third has decreased (Somer's d = -0.252; *P *<0.001)(Figure 2). The trend of location was seen in both sexes; and it was approximately equal (Somer's d: -0.250 in males and -0.263 in female; both *P *< 0.001)(figure 3). This increasing trend did not vary by age (under 50 versus upper 50) but was more pronounced among the younger (Somer's d: -0.239 in youngers and -0.227 in elders; both *P *< 0.001) (figure 4).

## Discussion

Adenocarcinoma of the upper and middle third of stomach shows an increasing trend during the last 30 years in Iran. Gastric cancers are the most common malignant neoplasm among Iranians [3,4]. They are the first leading causes of cancer-related deaths in Iranian males and the second in Iranian females [3] and, despite progress in diagnosis and treatment they have still poor prognosis (five year survival less than 20%)[1]. This study is the first one that focusing on changing of location of gastric adenocarcinoma in Iran. This study had some limitations; the most important one is that our study was not a population-based study, because cancer registry system in Iran is not complete yet and designing a population-based study is impossible now. However we believe that our study has minimal selection bias because 1) The selected center for this study is the most important referral center in Iran, 2) The cases were collected for a period of 36 years from all over the country. The other limitation was the incomplete macroscopic description of reports, particularly among early cases, a fact which leads to uncertainty in defining location sub-classification of tumors.

The advantage of this study is that all the related pathology reports and hematoxylin and eosin sections were reviewed by two pathologists. Consequently the error of misclassification that is propounded for increasing incidence of cardia adenocarcinoma in some studies [6,31] was minimized.

**Figure 5:**
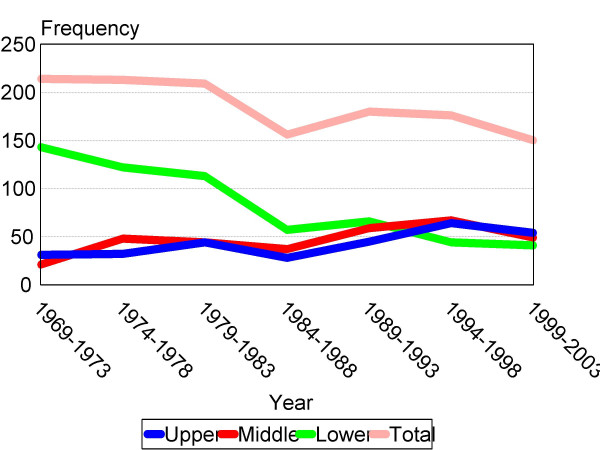
Total number of gastric adenocarcinoma per year was significantly decreased in recent 35 years and it was due to decreasing prevalence of lower third gastric adenocarcinoma. Spearman's rho: upper: 0.750 (*p *~ 0.05); middle: 0.750 (*p *~ 0.05); lower: -0.964 (*P *< 0.01)

Our study showed significant change in distribution of gastric adenocarcinoma as prevalence of carcinoma of upper and lower third reached from 15% in the first 10 years to 36% in the last 10 years of study period. One of the reasons that has usually been brought up for the increasing incidence of upper third gastric carcinoma is the decreasing incidence of lower third carcinoma due to of H. pylori eradication treatments leading to the relative increase of upper third carcinoma [32-37]. But this study showed not only decrease of prevalence of lower third adenocarcinomas but also independently increase of prevalence of upper and middle third adenocarcinoma (Spearman's rho: upper: 0.750 (*p *~ 0.05); middle: 0.750 (*p *~ 0.05); lower: -0.964 (*P *< 0.01))(Figure 5). Misclassification is another reason that has been brought up in some studies [7,31], but as will be shown later, this was limited in this study.

Disclosing of separate risk factors for cancers of upper third of stomach made this increasing incidence explanation available. Although many evidences show that treatment of H. pylori infection, especially cagA^+ ^strains can decrease incidence of distal gastric adenocarcinomas, several recent studies have noted that this treating might increase the risk of peptic esophagitis and adenocarcinoma of esophagus and cardia [32,37-39]. However some studies show that adenocarcinomas of distal esophagus are inversely associated with H. pylori but gastric cardia cancers have an unclear association with it [21,40-45]. Other risk factors that former studies have propounded are amount of dietary antioxidant [33], fruit and vegetable consumption [46], cigarette smoking [47,54] and obesity [53,55,56]. However in some reports gastric cardia cancers are only modestly associated with obesity [42] and are known to have no association with antioxidant intake [41]. In addition to the above risk factors Epstein – Barr virus (EBV) has been recently found to be associated with cardia tumors and it increases the risk of gastric cardia tumors five times [57]. Genetic factors are also important [58,59] and it has been shown that familial gastric cancers are frequently located in the cardia and are usually more aggressive than sporadic gastric cancers [60,61].

Urban development and industrialized living in recent decades in Iran have increased the probability of confrontation with much of the above risk factors, and this can be the cause of increasing prevalence of adenocarcinomas of upper third of stomach in this study. Decreasing prevalence of carcinoma of lower third of stomach can be due to early treatment of H. pylori infection in recent years and elimination of noxious diet. Cancers of middle third and lower third of stomach have *the *same risk factors; therefore we may normally expect a decreasing incidence of them. However our findings show an increasing prevalence of middle third gastric adenocarcinoma; the same was seen in another study in Japan [27]. We have no explanation for this and it needs more confirmatory studies.

In this study the trend of location was seen in both sexes, and it was approximately equal in both sexes. But Devesa's study in united state [6] and Botterweek's study in Slovakia, England, Wales and Scotland show the trends was more pronounced in females[25]. In other studies it was seen exclusively in males [12,16,27,62]. Less confrontation with risk factors such as smoking and other genetic and hormonal differences are propounded for explanation; however regarding of contradictory results this gender difference is largely unexplained and we could not find it [26].

With regard to small number of cases in our study an accurate evaluation of age effect in distribution of gastric cancers was impossible. However in a general analysis increasing prevalence of carcinomas of upper and middle third was a little more pronounced among the younger. This finding is against with other studies [6,27].

Generally increasing prevalence of upper third adenocarcinomas in this study is similar to increasing incidence seen in the United States [6,21,63,64], England [10,11], Denmark [12], the Netherlands [13], Sweden [14], New Zealand [15], Norway [16], Slovakia [17], Austria [18] and France [65]. It was not seen in all areas of the world. In two important population-based studies – Corley's [26] and Botterweek's [25] study – the results were not consistent in different areas and this can be due to different risk factors. There are also some contradictory results in the same areas. For example in Japan in one 30 year period study no change in gastric cancer location was seen [24], but in two other studies increasing incidences of gastric cardia cancer were seen [19,20].

Several studies have suggested that increasing incidence of proximal gastric cancers is seen in areas with low-risk for gastric cancer [23,24], but our findings in Iran which is one of the regions with high frequency did not show this change, similar to some other studies in Japan[19,20,27].

In conclusion, our findings show an increasing prevalence of proximal gastric cancers in our center during the last four decades. These findings express the changing in life style and so the risk factors; however we should consider other factors such as misclassification, and probability of selection bias in this study in regard of a few numbers of cases. Therefore we recommend a confirmatory population-based study. There are also hypothesis about histological trends in gastric adenocarcinoma, so in regard of lacking such a study in Iran our next step will be analysis of histological trend in gastric adenocarcinoma.

## Abbreviations

EBV: Epstein – Barr virus

## References

[B1] Jemel A, Murray T, Samuels A, Ghafoor A, Ward E, Thun M (2004). Cancer statistics, 2003. CA Cancer J Clin.

[B2] Noguchi Y, Yoshikawa T, Tsuburaya A, Motohashi H, Karpeh MS, Brennan MF (2000). Is gastric carcinoma different between Japan and United State?. Cancer.

[B3] Mohagheghi MA (2002). Final report of national research for cancer registration in Iran. National Cancer Registration of Iran.

[B4] Mossavi Jarrahi A, Mohagheghi M, Yazdizadeh B, Kolahi AA, Tahmasebi S, Sharifi K (2004). Analysis of smoking behavior in Iranian population: A cohort & period analysis. Asian Pacific Journal of cancer prevention.

[B5] Ekstrom AM, Hansson LE, Signorello LB, Lindgren A, Bergstrom R, Nyren O (2000). Decreasing incidence of both major histologic subtypes of gastric adenocarcinoma-a population-based study in Sweden. Br J Cancer.

[B6] Devesa SS, Blot WJ, Fraumeni JF (1998). Changing patterns in the Incidence of esophageal and gastric carcinoma in the United States. Cancer.

[B7] Devesa SS, Fraumeni JF (1999). The Rising incidence of gastric cardia cancer. Journal of the National Cancer Institute.

[B8] Antonio li DA, Goldman H (1982). Changes in the location and type of gastric adenocarcinoma. Cancer.

[B9] MacDonald WC, Mac Donald JB (1987). Adenocarcinoma of the esophagus and/or gastric cardia. Cancer.

[B10] Rios-Castellanos E, Sitas F, shepherd NA, Jewell DP (1992). Changing pattern of gastric cancer in Oxfordshire. Gut.

[B11] Harrison SL, Goldacre MJ, Seagrott V (1992). Trends in registered incidence of esophageal and stomach cancer in Oxford region, 1974–88. European Journal of cancer prevention.

[B12] Moller H (1992). Incidence of cancer of esophagus, cardia and stomach, in Denmark. European Journal of cancer prevention.

[B13] Van der Sanden GAC, Coebergh JWW, Vander Heijden LH, Verhagen TMT (1993). Trends in incidence of the gastrointestinal cancer in the southeastern Netherlands, 1975–1989. Tijdschr soc Gezondheidsz.

[B14] Hansson LE, Sparen Q, Nyren O (1993). Increasing incidence of carcinoma of the gastric cardia in Sweden from 1970 to 1985. British Journal of surgery.

[B15] Armstrong RW, Borman B (1996). Trends in incidence rates of adenocarcinoma of the esophagus and gastric cardia in New Zealand, 1978–1992. International Journal of epidemiology.

[B16] Hansens S, Wiig JN, Giercksky KE, Tretli S (1997). Esophageal and gastric carcinoma in Norway 1058–1992: incidence time trend variability according to morphological subtypes and organ subsites. International Journal of cancer.

[B17] Macfarlane GJ, Plesko I, Kramurova E, Obsitnikova A, Boyle P (1994). Epidemiological features of gastric and esophageal cancer in Slovakia. British Journal of cancer.

[B18] Thomas RJ, Lade S, Gilas GG, Tharsfield V (1996). Incidence trends in esophageal and proximal gastric carcinoma in victoria. Austria New Zeeland Journal of Surgery.

[B19] Kampschoer GH, NakaJina T, Van de Velde CJ (1989). Changing patterns in gastric adenocarcinoma. British Journal of surgery.

[B20] Blaser MJ, Saito D (2002). Trends in reported adenocarcinomas of the esophagus and gastric cardia in Japan. European Journal of gastroenterology and hepatology.

[B21] Blot WJ, Devesa SS, Kneller RW, Fraumeni JF (1991). Rising incidence of adenocarcinoma of the esophagus and gastric cardia. JAMA.

[B22] McKinney PA, Sharp L, Macfarlane GJ, Muir CS (1995). Esophageal and gastric cancer in Scotland 1960–1990. Br J Cancer.

[B23] Lee Jy, Kim Hy, Kim KH, Jang HJ, Kin JB, Lee JH (2003). No changing trends in incidence of gastric cardia cancer in Korea. Journal of Korean medical science.

[B24] Goto H, Ohmiya N, Kamiya K, Ando N, Sakata T, Hayakawa T (2001). Did gastric cancer vary over 30 years in Japan?. Gastroenterology.

[B25] Botterweek Anita Am, Schouten LJ, Volorics A, Orant E, Van den Brandt P (2000). Trends in incidence of adenocarcinoma of the esophagus and gastric cardia in ten European countries. International Journal of epidemiology.

[B26] Corley DA, Buffler PA (2001). Esophageal and gastric cardia adenocarcinomas: analysis of regional variation using the cancer incidence in five continents database. International Journal of epidemiology.

[B27] Liu Y, Kaneko S, Sobue T (2004). Trends in reported incidences of gastric cancer by tumor location, from 1975 to 1989 in Japan. International Journal of epidemiology.

[B28] Japanese Gastric Cancer Association (1999). Japanese classification of gastric carcinoma.

[B29] Misumi A, Murakami A, Harada K, Akagi M (1989). Definition of carcinoma of the gastric cardia. Langenbecks Arch Chir.

[B30] Owen DA, Mills SE, Catter D, Greenson JK, Oberman HA, Reuter V, Stoler MH (2004). The stomach. Sternberg's Diagnostic Surgical Pathology.

[B31] Ekström AM, Signorello LB, Hansson LE, Bergstrom R, Lindgren A, Nyren O (1999). Evaluating gastric cancer misclassification: a potential explanation for the rise in cardia cancer incidence. Journal of the National Cancer Institute.

[B32] Chow WH, Blaser MJ, Blot WJ, Gammon MD, Vaughan TL, Risch HA (1998). An inverse relation between cagA^+ ^strains of helicobacter pylori infection and risk of esophageal and gastric cardia adenocarcinoma. Cancer Res.

[B33] Erkisi M, Colakoglu S, Koksal F (1997). Relationship of Helicobacter Pylori infection to several malignant and non malignant gastrointestinal diseases. J Exp Clin Cancer Res.

[B34] Ekstrom AM, Held M, Hansson LE, Engstrand L, Nyren O (2201). Helicobacter Pylori in gastric cancer established by CagA^+ ^immuniblot as marker of past infection. Gastroenterology.

[B35] Hunt RH, Sumanac K, Huang JQ (2001). Review article: Should we kill or should we save Helicobacter Pylori?. Aliment Pharmacol Ther.

[B36] Correa P, Shiao YH (1994). Phenotypic and genotypic events in gastric carcinogenesis. Cancer Res.

[B37] Hansen LR, Engstrand L, Nyren O, Lindgren A (1995). Prevalence of Helicobacter pylori infection in subtypes of gastric cancer. Gastroenterology.

[B38] de Koewin JD (2001). Eradication of Helicobacter Pylori. Is it necessary to eradicate Helicobacter Pylori in gastric reflux?. Press Med.

[B39] Kandel G (2000). Helicobacter and disease: still more question than answers. Can J Surg.

[B40] Legergren J, Bergstrom R, Adami HO, Nyren O (2000). Association between medication that relax the lower esophageal sphincter and risk for esophageal adenocarcinoma. Ann Int Med.

[B41] Terry P, Lagergren J, Ye W, Nyren O, Wolk A (2000). Antioxidants and cancers of the esophagus and gastric cardia. Int J Cancer.

[B42] Lagergren J, Bergstrom R, Nyren O (1999). Ann Intern Med.

[B43] Spechler SJ (1999). The role of gastric carditis in metaplasia and neoplasia at the gastroesophagial junction. Gastroenterology.

[B44] Pera M (2000). Epidemiology of esophageal cancer, especially adenocarcinoma of the esophagus and esophagogastric junction. Recent Results Cancer Res.

[B45] Limburg P, Qiao Y, Mark S (2001). Helicobacter Pylori seropositivity and subsite-specific gastric cancer risks in Linxian, China. J Natl Cancer Inst.

[B46] Terry P, Lagergren J, Hansen H, Wolk A, Nyren O (2001). Fruit and vegetable consumption in the prevention of esophageal and cardia cancers. Eur J Cancer Prev.

[B47] Sasazuki S, Sasaki S, Tsugane S (2002). for the Japan Public Health Center Study Group. Cigarette smoking, alcohol consumption and sub sequent gastric cancer risk by subsite and histologic type. Int J Cancer.

[B48] Gammon MD, Schoenberg JB, Ahsan H, Risch HA, Vaughan TL, Chow WH (1997). Tobacco, alcohol, and socioeconomic status and adenocarcinomas of the esophagus and gastric cardia. J Natl Cancer Inst.

[B49] Gray JR, Coldman AJ, MacDonald WC (1992). Cigarette and alcohol use in patients with adenocarcinoma of the gastric cardia or lower esophagus. Cancer.

[B50] Kabat GC, Ng SK, Wynder EL (1993). Tobacco, alcohol intake, and diet in relation to adenocarcinoma of the esophagus and gastric cardia. Cancer Causes Control.

[B51] Zhang ZF, Kurtz RC, Sun M, Karpeh MJR, Yu GP, Gargon N (1996). Adenocarcinomas of the esophagus and gastric cardia: medical conditions, tobacco, alcohol, and socioeconomic factors. Cancer Epidemiol Biomarkers Prev.

[B52] Brown LM, Silverman DT, Pottern LM, Schoenberg JB, Greenberg RS, Swanson GM (1994). Adenocarcinoma of the esophagus and esophagogastric junction in withe men in the United States: alcohol, tobacco, and socioeconomic factors. Cancer Causes Control.

[B53] Vaughan TL, Davis S, Kristal A, Thomas DB (1995). Obesity, alcohol, and tobacco as risk factors for cancers of the esophagus and gastric cardia: adenocarcinoma versus squamous cell carcinoma. Cancer Epidemiol Biomarkers Prev.

[B54] Garidou A, Tzonou A, Lipworth L, Signorello LB, Kalapothaki V, Trichopoulos D (1996). Life-style factors and medical conditions in relation to esophageal cancer by histologic type in a low-risk population. Int J Cancer.

[B55] Chow WH, Blot WJ, Vaughan TL, Risch HA, Gammon MD, Stanford JL (1998). Body mass index and risk of adenocarcinomas of the esophagus and gastric cardia. J Natl Cancer Inst.

[B56] Brown LM, Swanson CA, Gridley G, Swanson GM, Schoenberg JB, Greenberg RS (1995). Adenocarcinoma of the esophagus: role of obesity and diet. J Natl Cancer Inst.

[B57] Corvalan A, Koriyama C, Akiba S (2001). Epstein-Barr virus in gastric carcinoma is associated with location in the cardia and with a diffuse histology: a study in one area of Chile. Int J Cancer.

[B58] Lee KH, Lee JS, Suh C (1995). Clinicopathologic significance of the K-ras gene codon 12 point mutation in stomach cancer. An analysis of 140 cases. Cancer.

[B59] Shen H, Xu Y, QIen Y (2000). polymorphisms of the DNA repair gene XRCC1 and risk of gastric cancer in a Chinese population. Int J Cancer.

[B60] Kakiuchi H, Itoh F, Kusano M (1999). familial gastric cancer in in the Japanese population is frequently located at the cardiac region. Tumor Biol.

[B61] Liu Y, Yushimura K, Yamaguchi N, Shinmura K, Yokota J, Katai H (2003). Causation of Burrmann type 4 gastric cancer: heritable factors or environmental factors?. Gastric cancer.

[B62] Yang PC, Davis S (1988). Incidence of cancer of esophagus in the US by histologic type. Cancer.

[B63] Correa P, Chen VW (1994). Gastric cancer. Cancer Surv.

[B64] Yang PC, Davis S (1988). Epidemiological characteristics of adenocarcinoma of the gastric cardia and distal stomach in the United States, 1973–1982. Int J Epidemiol.

[B65] Liabeuf A, Faivre J (1997). Time trends in esophageal cancer incidence in Cote d'Or (France), 1976–93. Eur J Cancer Prev.

